# Determinants of Bone Mineral Screening Behavior among Three Ethno-Cultural Groups of Women in Israel

**DOI:** 10.3390/ijerph17176138

**Published:** 2020-08-24

**Authors:** Offer E. Edelstein, Netta Achdut, Iris Vered, Orly Sarid

**Affiliations:** 1 Spitzer Department of Social Work, Ben-Gurion University of the Negev, Be’er Sheva 8410501, Israel; nettaach@bgu.ac.il (N.A.); orlysa@bgu.ac.il (O.S.); 2 Division of Endocrinology, Diabetes and Metabolism, The Chaim Sheba Medical Centre, Tel Hashomer, Ramat Gan 5262000, Israel; Iris.Vered@sheba.health.gov.il

**Keywords:** BMD, ethno-cultural background, TPB, knowledge

## Abstract

Bone mineral density (BMD) screening is one of the main means to detect and treat osteoporosis. Yet, the manner in which ethno-cultural background is associated with BMD health cognitions and screening behavior remains limited. Several ethno-cultural groups (*n* = 100 in each group)—Israeli-born Jews, Israeli-born Bedouin-Muslims, and Jewish immigrants from the Former Soviet Union (FSU), mean age 70 (SD = 7.1)—participated in face-to-face interviews in a cross-sectional survey, using valid and reliable questionnaires on BMD screening behavior, knowledge about osteoporosis, and theory of planned behavior (TPB) components. FSU immigrants reported the lowest BMD screening behavior. The multivariate analysis showed that higher knowledge level, positive attitudes, supportive subjective norms, and greater intentions increase the probability of BMD screening behavior. The TPB attitude component had a more pronounced effect on the probability of undergoing BMD screening among Israeli-born Bedouin-Muslims compared to Israeli-born Jews. Our findings contribute to the TPB by deepening our understanding of the associations between TPB components and BMD screening behaviors, from an ethno-cultural perspective. To assure sufficient BMD screening behavior among all ethno-cultural groups, intervention programs—suited to address the unique characteristics of each ethno-cultural group—are required.

## 1. Introduction

According to the definition of the World Health Organization (WHO), osteoporosis is “a progressive systemic skeletal disease characterized by low bone mass and micro-architectural deterioration of bone tissue, with a consequent increase in bone fragility and susceptibility to fracture” [1]. It is estimated that, by 2025, more than 30 million European women and men aged 65 years and above will be affected by osteoporosis [2]. Other estimates show that approximately 53 million individuals either are at high risk for osteoporosis or already have it [3]. The lifetime risk for fractures varies between women and men and in accordance with the location of the fracture: hip (23% and 11%, respectively), spine (29% and 14%, respectively), and wrist (21% and 5%, respectively) [4].

Osteoporosis and its related fractures bear a substantial societal economic burden [5,6,7], and they are often associated with diminished quality of life [8], greater disability [9], morbidity [10], and increased risk for mortality, independent of age [11]. This disease is regarded as “silent”, due to an absence of symptoms before the occurrence of fractures [12]. Bone mineral density (BMD) screening, performed using dual-energy X-ray absorptiometry (DXA), is one of the main measures used to detect and treat bone density deterioration [13]. This screening is recommended by health promotion agencies [14,15,16] for all women aged 65 or above (senile osteoporosis), as well as for postmenopausal women younger than 65 years, who are at increased risk of osteoporosis. Nevertheless, osteoporosis remains an underdiagnosed and undertreated condition [16]. For example, United States (US) estimates demonstrated substantial deficiencies in performance of BMD screenings among older women, ranging from 12.8% among women aged 80+ to a higher percentage among women aged 50–64 and 65–79 (21.1% and 26.5%, respectively) [17]. Low performance of BMD screenings was found in other countries such as Denmark [18], Australia [19], and Israel [20].

In attempts to understand disparities concerning BMD screening behavior, several studies found that socio-demographic variables such as lower education level and socioeconomic status are associated with lower performance rates [17,18]. These socio-demographic variables were also associated with a lower likelihood of participation in other health-related behaviors among older persons, such as physical activity [21,22] and calcium intake [23,24,25,26].

Considerable research attention was given to exploring the cognitive variables that might be associated with participation and adherence to health-related behaviors [27,28,29]. One of the widely used theoretical frameworks for understanding disparities in health behaviors is the theory of planned behavior (TPB) [30]. *Behavioral intent* is a key component of TPB, and it precedes an actual behavior [30]. A plethora of studies about participation in health behaviors such as physical activity [28,31,32], diet [33], and eating behaviors [34] supported the prominent role of behavioral intent among adults. TPB further elaborates on three components that might influence behavioral intent [30]: *attitude,* which reflects an individual’s favorable or unfavorable appraisals about a particular behavior and consideration of the outcomes of performing it; *subjective norms*, which include perceptions about the acceptability attributed to a certain behavior by peers, significant others or cultural context; *perceived behavioral control,* which comprises individual perceptions about the extent of difficulty associated with the performance of the behavior. Despite the fact that behavioral intent is at the core of TPB [30], other studies suggest that TPB components (i.e., attitudes, subjective norms, perceived behavioral control) directly influence participation in physical activity [35,36], weight-loss behaviors [37], and adherence to prescription drugs [38]. To our knowledge, the current study is the first to examine the TPB model in relation to predicting BMD screening behavior.

Health disparities are associated with ethno-cultural background and can be depicted by participation in health-related behaviors. In the United States, Gillespie and Morin [17] found that older African-American women, compared to other ethno-cultural groups, were least likely to undergo BMD screening. Colorectal cancer screening disparities were observed among Asian Americans, compared to non-Latino Caucasians [39]. Further ethno-cultural-based disparities were found between African-American and Caucasian women in receiving adjuvant chemotherapy and radiotherapy for breast cancer [40]. Israeli studies showed that the use of prescribed medications was lower among Jewish immigrant women from the Former Soviet Union (FSU) [41], compared with Israeli-born women. Other studies found that Jewish FSU immigrants and Arab women reported a lower number of preventive visits and screening tests (i.e., mammogram, Papanicolaou (Pap) smear, and bone density) compared to Israeli-born Jewish women) [20].

Ethno-cultural background might be associated not only with a certain behavior practice, but also with ethno-cultural appraisals about behavioral adequacies [42]. In this instance, Hofstede [43] stated that entities such as cognitions, attitudes, and internalization of social norms can be viewed differently across cultures. For example, cultures differ regarding the significance attached to individualist vs. collectivist values and norms. Masculine vs. feminine notions are another continuum along which gender roles and the behaviors derived from these roles can be studied [44]. This conceptualization forms the basis of our hypothesis that women from a conservative and patriarchal society tend to adhere to traditional gender roles and subscribe to the point of views of authoritative (male) figures (e.g., Bedouin Muslims) compared to Israeli-born Jewish women, who are raised in a more Western, individualistic culture [45,46,47]. More specifically related to osteoporosis, the findings of a recent study conducted in Israel [28] showed that Bedouin-Muslim women reported the lowest engagement in physical activity, accompanied by less supportive norms or favorable attitudes toward the subject.

Lastly, research attention was given to the associations between knowledge level and participation in health behaviors. In general, there is conflicting evidence about the role of knowledge in the prediction of health behaviors. Several studies found that a higher level of knowledge was associated with greater participation in health behaviors such as physical activity [27], calcium intake, and vitamin D dietary consumption [48,49,50]. However, other studies did not provide support for such associations [51,52,53,54].

In the current study, we examined the underlying factors, suggested by the TPB model [30], associated with BMD screening behavior among older women from three ethno-cultural groups in Israel: Israeli-born Jews, Israeli-born Bedouin-Muslims, and Jewish immigrant women born in the Former Soviet Union (FSU). Using a multi-cultural perspective, we sought to (i) assess the differences among the three ethno-cultural groups (Israeli-born Jews, Israeli-born Bedouin-Muslims, and Jewish immigrants from the FSU) in regard to knowledge about osteoporosis, TPB components related to BMD screening, and screening behavior, and (ii) to construct a model for estimating BMD screening behavior, comprising socio-demographic variables, ethno-cultural background, knowledge level, and TPB components.

We hypothesize that (1) the ethno-cultural groups will differ regarding their knowledge about osteoporosis and the TPB components related to the BMD screening behavior, (2) BMD screening behavior will be associated with ethno-cultural background, knowledge, and TPB components, and (3) the effect of knowledge and TPB components on BMD screening behavior will vary according to ethnicity.

## 2. Method

### 2.1. Participants

Using quota sampling, a total of 300 women participated in the study (100 from each ethno-cultural group). Inclusion criteria were as follows: all participants had to (a) pertain to one of the three ethno-cultural groups, (b) be 60 years of age or above, and (c) be able to complete the questionnaire in their mother tongue. According to Fleiss, Levin, and Paik [55], a sample of 300 respondents was required. Two-sided significance levels were calculated (1-alpha; 0.95), with an 80% power level (1-beta), meaning there was an 80% chance of detecting ethno-cultural differences concerning health attitudes and behaviors (for example, see Sarid et al. [45]). The statistical power of the proposed sample size was confirmed using Gpower 3 software. We approached 358 women. A total of 27 potential participants refused to sign the informed consent form. An additional 31 failed to complete the questionnaire and, as a result, were excluded. These women pertained to all three groups. The total response rate reached 83.7% (300/358).

### 2.2. Measures

#### 2.2.1. Dependent Variable

*BMD screening behavior* was assessed by a self-reported single item indicating whether the participant underwent a bone screening in the past year (no/yes).

#### 2.2.2. Independent Variables

*Socio-demographic characteristics*: Our main demographic variable was the participant’s ethno-cultural background: Israeli-born Jew, immigrant from the FSU, and Israeli-born Bedouin-Muslim. For this variable, we created two dummy variables with the reference category of Israeli-born Jews. Further socio-demographic data included the following: age (in years); marital status (single/married or cohabitating/divorced/widow). We created a dichotomous dummy variable where married or cohabitating received the value of 1 (other = 0); the number of children was also determined. Education level was recorded according to the International Standard Classification of Education [56] and divided into low (less than lower secondary education, or lower/secondary education completed), medium (upper secondary education or post-secondary non-tertiary education completed), and high (tertiary education completed). Dummy variables representing three categories were used in the analyses, with low education serving as the reference category: employment status (woman engaged in paid work received the value of 1). Perceived financial situation was measured by a single item: “how would you describe your family’s financial situation?” Response choices were “well above average”, “somewhat above average”, “about average”, “somewhat below average”, and “well below average”. Due to the skewed distribution of responses, the first two categories were combined to form an above average category, and the latter two categories were combined to form a below average category for analysis purposes. Dummy variables representing three categories were used in the analyses, with below average serving as the reference category. Similar subjective measures of financial situation were used in prior health studies [57,58]. Degree of religiosity was measured using a dichotomy dummy variable where traditional and religious participants received the value of 1 (secular = 0).

*Knowledge regarding osteoporosis* was assessed by an adapted version of the 24-item Facts on Osteoporosis Quiz (FOOQ) [59]. Each item can be answered dichotomously. Similar to previous studies [27,28], we added a “do not know” response to reduce the likelihood of guessing. The “do not know” response was coded as 0. An index was calculated by summing the correct responses (coded as 1), ranging from 0 to 24, with a higher score indicating a higher level of osteoporosis-related knowledge. The internal consistency for this study was good (Cronbach’s α = 0.73).

In this study, TPB component measurements were largely based on those suggested by Fishbein and Ajzen [60], referring to each TBP component. Following more recent studies on health behaviors, which used TPB as a theoretical framework [61,62,63] in our study, the components were assessed as follows:

*Perceived behavioral control* was assessed by two items: “I am confident that I can undergo a BMD screening”, and “undergoing a BMD screening depends on my will “. Each item was scored on a scale ranging from 1 = “disagree” to 7 = “agree”. An index summing the items was calculated, and it ranged from 2–14. The internal consistency for this study was good (Cronbach’s α = 0.79).

*Attitudes* were assessed using two items: “undergoing a BMD screening will be…”, where one item was scored 1 = “bad for me” to 7 = “good for me”, and the other item ranged from 1 = “very unpleasant for me” to 7 = “very pleasant for me”. An index summing the items was calculated, and it ranged from 2–14. The internal consistency for this study was satisfactory (Cronbach’s α = 0.69).

*Subjective norms* were assessed by two items: “most people who are meaningful to me support BMD screening behavior “, and “most people in my condition regularly undergo BMD screening”. Each item was scored on a scale ranging from 1 = “disagree” to 7 = “agree”. An index summing the items was calculated, and it ranged from 2–14. The internal consistency for this study was good (Cronbach’s α = 0.71).

*Intention to undergo BMD screening* was assessed by a single item: “I intend to undergo BMD screening”. This item was scored on a scale ranging from 1 = “disagree” to 7 = “agree”.

### 2.3. Procedure

The questionnaires were back-and-forth translated from Hebrew into Arabic and Russian by four independent bilingual professional translators (two for each language). Before interviewing, we performed a pretest of 10 questionnaires for each group (*n* = 20) to evaluate item comprehension and style correction. In order to assure high participation rates, we recruited qualified interviewers who speak Arabic, Russian, or Hebrew at the mother-tongue level. The interviewers approached potential participants near local community health clinics and centers. The aims of the study were explained, and participants who expressed interest signed an informed consent form. Participants underwent face-to-face interviews at a time and place of their choice. Each interview lasted for about half an hour and there was no compensation for participation. The study protocol was approved by the Ethics Committee at Ben-Gurion University of the Negev.

### 2.4. Data Analysis

Bivariate analysis was firstly carried out to examine differences in knowledge, as well as the TPB components of perceived control, attitudes, subjective norms, and behavioral intent according to ethno-cultural background. Secondly, we conducted a bivariate analysis to assess the associations between the dependent variable—BMD screening behavior in the past year (yes/no), and the following variables: demographic characteristics, knowledge about osteoporosis, TPB model components, and behavioral intent. Chi-square tests for heterogeneity and trend were calculated for categorical variables. ANOVA (with Bonferroni post hoc comparisons) and *t*-tests were computed in order to assess differences in means of knowledge level, TPB components, and intentions.

Thirdly, in order to estimate a model for the prediction of BMD screening behavior in the past year, we employed five hierarchical logistic models. Model 1 included the demographic characteristics (detailed in Table 1). Religiosity was excluded in the model, due to small cell constraints. We also excluded indication of employment status, due to multicollinearity with the variable of “age” (Spearman correlation = −0.512) (Model 1). In Model 2, knowledge about osteoporosis was added (Model 2), followed by the TPB model components in Model 3. In Model 4, based on the theoretical structure of TPB [30], we added the behavioral intention variable (Model 4). To test whether the effect of the knowledge and TPB components—perceived control, attitudes, subjective norms, and intention—varied among the three ethno-cultural groups, we used two-way interaction terms between ethno-cultural groups and the knowledge/TPB components. However, since we considered five factors (the knowledge variable + four TPB components) and three groups—yielding a total of 10 interaction terms—and due to multi-collinearity, we could not include all of the interaction terms in a single model. Thus, we estimated our model with two interaction terms at a time (immigrants × TPB component and Bedouin-Muslims × TPB component). The same was applied regarding knowledge (immigrants × knowledge and Bedouin-Muslims × knowledge). To avoid cumbersome and unnecessary repetitions, we present one model in which the interaction terms were significant (Model 5). Estimations derived from models including non-significant interaction effects are available upon request. The dependent variable in all analyses was “underwent BMD screening behavior in the past year (=1)”. Data were analyzed using IBM SPSS ver. 25 (IBM Corporation, Armonk, NY, USA) Statistics for Windows.

## 3. Results

Table 1 summarizes the descriptive statistics of the study variables in the overall sample and according to ethno-cultural group. We found considerable ethno-cultural-based significant differences for most of the socio-demographic variables. Israeli-born Jews comprised the youngest group (mean = 68.2 (6.4)), while Israeli-born Bedouin-Muslims made up the oldest (mean = 74.2 (6.8)).

Israeli-born Bedouin-Muslims had more children compared to the other two groups and were more likely to report being traditional or religious (98.9%) than FSU Jewish immigrants (23.3%) and Israeli-born Jews (71.1%). Higher education was more prevalent among FSU Jewish immigrants (51%), while a low level of education was reported by the Bedouin-Muslims (100%). Surprisingly, a higher percent of Bedouin-Muslims reported their financial situation as being “above average” (50%) compared to FSU Jewish immigrants (11.2%) and Israeli-born Jewish participants (27.3%). Employment was more prevalent among both Israeli-born Jews (46.5%) and FSU Jewish immigrants (37.4%) than among Israeli-born Bedouin-Muslims (3%).

Compared to the other two ethno-cultural groups, Bedouin-Muslims scored significantly low on the following indices: knowledge level, perceived control, attitudes, subjective norms, and intention to undergo BMD screening (Table 2). For instance, the mean score of attitudes toward BMD screening was 9.64 (3.06) among Israeli-born Bedouin-Muslims, compared to the higher mean scores found among both Israeli-born Jews (11.74 (2.39)) and FSU Jewish immigrants (11.43 (3.04)). Israeli-born Jews reported higher perceived control, compared to FSU Jewish immigrants (13.14 (1.85) and 11.88 (3.09), respectively). No other statistically significant differences were found between Israeli-born Jews and FSU Jewish immigrants.

Table 3 presents a bivariate analysis of the study variables by BMD screening behavior. Older participants with a traditional or religious orientation, those who subjectively assessed their financial situation as “above average”, and women not engaged in paid work were more likely to undergo BMD screening. Israeli-born Jews reported the highest BMD screening behavior (50.5%), while the lowest was reported by FSU Jewish immigrants (33.3%). Israeli-born Bedouin-Muslims reported similar performance (48.2%) to that of Israeli-born Jews. Statically significant differences in BMD screening behavior were further observed for the following TPB components: perceived control, attitudes, subjective norms, and intention. To elaborate, the mean score of attitudes toward performing BMD screening was 11.89 (1.99) among participants who underwent the screening versus 10.40 (3.35) among participants who did not. Similarly, the mean score of the subjective norms was higher among participants who underwent BMD screening (11.16 (2.46)) versus those who did not (9.53 (3.43)).

Table 4 displays five hierarchical logistic models for predicting BMD screening behavior. According to Model 1, older participants (odds ratio = 1.11), those who were married or cohabitating (odds ratio = 2.65), and those who reported an “above average” financial situation (odds ratio = 1.27) were more likely to undergo BMD screening. FSU Jewish immigrants were less inclined to undergo BMD screening compared to Israeli-born Jews (odds ratio = 0.43) and Israeli-born Bedouin-Muslims (odds ratio = 0.27). No differences were found among the groups for the variables number of children and education level. In Model 2, greater knowledge of osteoporosis was positively associated with the probability of undergoing BMD screening (odds ratio = 1.10). Adding the TPB components (Model 3) showed that favorable attitudes and supportive subjective norms about BMD screening increased the probability of screening behavior (odds ratio = 1.21 and odds ratio = 1.14, respectively). Perceived control regarding BMD screening had no effect on the probability to undergo screening. Moreover, the differences in the probability to undergo screening according to ethnicity diminished once the TPB components were added to the model. This suggests that the attitudes and subjective norms components fully mediated the association between ethno-cultural background and BMD screening behavior. Model 4 indicated that greater intention to undergo BMD screening greatly increased the probability of doing so (odds ratio = 1.92) (Model 4). Finally, the last model (Model 5) included two-way interaction terms between ethno-cultural origin and attitudes toward BMD screening. We found that the effect of the attitudes component was more pronounced among Israeli-born Bedouin-Muslims than among Israeli-born Jews. No such effect was found among FSU Jewish immigrants, suggesting that the attitudes component uniformly affects BMD screening behavior among both FSU Jewish immigrants and Israeli-born Jewish participants. All other interaction terms tested in association with ethno-cultural groups and TPB components, knowledge, and intention were statistically insignificant (not shown). This indicates that the two minority groups were not found to be more or less sensitive to the model components than the majority group.

## 4. Discussion

The first aim of the study was to assess the differences among the three ethno-cultural groups in regard to knowledge about osteoporosis, TPB components related to BMD screening, and screening behavior. We hypothesized that ethno-cultural groups will differ in regard to their knowledge about osteoporosis and TPB components related to performing BMD screening. The findings of the current study indicate low levels of knowledge among all participants, with Israeli-born Bedouin-Muslims scoring the lowest. Previous studies corroborate our findings on lack of knowledge about osteoporosis, as well as the existence of significant gaps among minority groups [28,64]. Israeli-born Bedouin-Muslims reported very low levels of education and were less likely to be employed. These characteristics are assumed to be associated with limited and weaker social networks, and possibly with fewer opportunities to be exposed to knowledge and information, including health-related knowledge and information, transferred by social networks [65,66]. The importance of an extensive social network, as an agent that may enhance various health behavior-related practices, was studied previously [67].

Our findings indicate that about half of Israeli-born Jews and Israeli-born Bedouin-Muslims reported preforming BMD screening, while only one-third of the FSU Jewish immigrants reported doing so. Previous studies found that long-term Israeli Jewish female residents reported a higher level of screening behaviors (including BMD screenings) than FSU Jewish immigrants and Israeli Arab women [20]. Hence, our findings are consistent with the differences found between long-term Jewish residents and FSU Jewish immigrants in Israel; however, they do not provide further support for the low BMD screening rates reported in previous studies among Israeli Arabs. A possible explanation for this discrepancy is the fact that, contrary to previous studies, our sample includes only Israeli-born Bedouin-Muslims, a subgroup of the Arab society in Israel. Previous studies among Israeli Bedouin-Muslims showed that mothers had the highest mean rate of vaccination behavior compared to Israeli-born Jewish or FSU Jewish immigrant mothers [45]. The similar rates of BMD screening reported by Israel-born women (Jewish and Muslims) may actually stem from different—and even opposite—cultural orientations. For example, it is possible that Israeli-born Jewish participants express a more individualistic way of taking care of themselves [45]. It is also possible that the high levels of sense of control and attitudes among Jewish women reported in the current study reflect their stronger individualistic orientation compared to the other groups. Israeli-born Bedouin-Muslims, on the other hand, might perform BMD screenings in response to their collectivistic masculine cultural orientation, which highlights the importance of obeying authority figure recommendations, including medical regulations and referrals from health professionals [45,68]. Furthermore, we found that FSU Jewish immigrants resemble the Israeli-born Jewish participants in most of the TPB components, apart from lower levels of perceived control toward the screening and lower performance rates. The current findings provide further support for the influence of the prolonged nature of acculturation processes on health behaviors; some cognitions toward health behaviors might change as time in the new country passes, while others do not [45]. It is possible that immigrants’ lower level of perceived control might be related to social barriers such as language proficiency [69,70], resulting in lower actual levels of preventive health behaviors.

The second aim of the current study was to examine the contribution of socio-demographic characteristics, with ethno-cultural background as the focus, along with knowledge level and TPB components, in explaining BMD screening behavior. We hypothesized that BMD screening behavior will be associated with ethno-cultural background, knowledge, and TPB components. We found substantial variations in the probability to perform BMD screening according to ethno-cultural background. Israeli-born Jews were far more likely to report this health behavior than the other groups. Contrary to the bivariate analysis, our multivariate analysis revealed that Israeli-born Bedouin-Muslims were less likely to undergo BMD screening, compared to Israeli-born Jews; this is after controlling for demographic characteristics and knowledge about osteoporosis. Our findings support the results of previous studies of health disparities among minorities related to preventive screening [20,71] and medical care usage [41]. Moreover, in line with other studies that demonstrated the associations between knowledge level and participation of older adults in health behaviors such as calcium intake [48,49], vitamin D consumption [48], and adults’ BMD screening [50], our findings indicate that higher knowledge about osteoporosis increased the odds of women to undergo the screening.

According to the Israeli National Health Insurance Law, all citizens of Israel are entitled to comprehensive healthcare services [72]; however, inequality in terms of availability, accessibility, and, in some cases, even the quality of these services still exist. It might be that Israeli-born Bedouin-Muslims, many of whom reside in geographically remote areas in the southern part of Israel, are subjected to limited healthcare services, particularly with regard to specialists, due to distance, as well as a shortage of physicians and other health professionals [73]. Yet, the accessibility of services is not the only possible explanation for the relatively low rates of BMD screening behavior. Lack of knowledge among Israeli-born Bedouin-Muslims, compared to other ethno-cultural groups, may also influence health behavior [28,45]. Thus, health disparities are not necessarily related to the provision of healthcare services, but rather to the lack of knowledge about them. This explanation, however, might not apply to FSU-born Jewish women, who live in all parts of Israel and are not subjected to geographical-based deficits. As mentioned earlier, we believe that disparities in health behaviors between Israeli-born Jewish and FSU-born Jewish women are mainly due to social and cultural differences related to mistrust concerning governmental health agencies [45].

As for the contribution of TPB components to BMD screening behavior, our findings generally support the TPB theoretical concepts [30], indicating that, everything else being equal, more positive attitudes and supportive subjective norms increase the probability of performing the behavior in question, i.e., BMD screening. Although our bivariate analysis showed differences in the probability to undergo BMD screening by perceived control, the multivariate analysis did not confirm such an association. This runs counter to the theoretical assumption that greater perceived control will be associated with an actual health behavior [30]. Perceived control plays an important role in predicting a health behavior that is routinely performed, such as physical exercise [36] and diet [74], where one’s self-persistence and motivation are central. Our findings suggest that BMD, a type of screening that is performed only occasionally, might resemble other preventive behaviors such as breast cancer screening, where subjective norms and personal attitudes [75] are more prominent determinants of such behaviors.

Moreover, we found that greater intention to undergo BMD screening increased the probability of reporting doing so. This finding is consistent with the main assumption of the TPB model [30] and corroborates the findings of numerous studies on health behaviors [28,31,32,33,34], thus highlighting the centrality of behavioral intention in explaining actual behavior.

Our third hypothesis was that the effect of knowledge and TPB components on BMD screening behavior will vary according to ethnicity. We found limited evidence of a moderation effect, as tested between ethno-cultural background and TPB components, with one exception; the effect of the attitude component on the probability to perform BMD screening was more pronounced among Israeli-born Bedouin-Muslims than among Israeli-born Jews. That is, the link between attitudes and BMD screening behavior is stronger among Israeli-born Bedouin-Muslims women than among Israeli-born Jewish women. A possible explanation for this finding is the importance placed on a woman’s personal attitude compared to the internalization of social norms (subjective norms) in adopting a certain behavior. Contrary to Israeli-born Jewish women who may express their health cognitions through the different aspects assessed by the TPB model, Muslim women, who belong to the collectivist, male-dominated Bedouin culture, might place greater importance on their own quiet voice (as portrayed by the attitude aspect), instead of subscribing to the internalization of social norms, as a way to convey their preference of personal health behaviors. This explanation underpins the substantial changes that the Bedouin-Muslim society experienced in recent years [76,77], such as opening up to more Western ideologies and practices. Thus, although the role of authoritative figures in this society is still prominent [76], there is more of an opportunity for the individual to express some degree of personal attitudes and preferences. It is also possible that, compared to Israeli-born Bedouin-Muslims, Israeli-born Jews have a higher compatibility level between attitudes and social norms, due to a more coherent internalization of social norms related to the necessity to perform BMD screening in order to promote women’s health. Prior studies demonstrated that cultural-based differences in attitudes and subjective norms about certain behaviors might derive from cultural pragmatism about the behavior in question [78]. All other interaction terms tested among the three ethno-cultural groups and TPB components, knowledge, and intention were found to be statistically insignificant, indicating that the associations linking knowledge, perceived control, subjective norms, and intentions to undergo BMD screening appear to be uniform. These findings reinforce the applicability of the TPB model for explaining health behaviors as a general model [79], suited to diverse ethno-cultural backgrounds.

Health promotion is a community task, involving many partners, including one’s immediate social networks, agencies, and healthcare system (accessibility and availability of services). Therefore, we encourage researchers to formulate extended theoretical conceptualizations to enhance our ability to integrate environmental and individual variables contributing to the promotion of specific health behaviors. It can be interesting to study TPB components in a broader context, for example, that of ecological system theory [80]. To support this type of extended theoretical conceptualization, future studies should collect data on TPB components among spouses, siblings, health professionals, and additional significant others surrounding the women, vis à vis a certain health behavior, along with indicators reflecting the accessibility and availability of healthcare services for women, for example, living in different geographical areas.

## 5. Limitations

The current study, together with its merits, has several limitations. Firstly, the use of quota sampling limits our ability to generalize the findings to other minority groups. We encourage other researchers to conduct studies using representative samples. Secondly, our study focused on the role of individual-level indicators in shaping actual health behaviors, while other social and environmental factors assumed to promote or impede personal health behaviors were not examined [81]. Thirdly, BMD screening behavior was self-reported by the participants. Future studies should consider corroborating self-reported behaviors with objective means, such as medical records. Fourthly, we used face-to-face interviews, which may have increased the likelihood of social desirability bias in measurements of attitudes and behaviors [82,83].

## 6. Conclusions

Our findings contribute to the TPB model by providing a deeper understanding of the association among ethno-cultural background, TPB components, and a specific preventive health behavior—BMD screening. With respect to the theoretical implications, our findings demonstrated that TPB components had a differential weight in explaining elderly women’s behavior regarding a type of preventive screening, which is performed only occasionally. The association between Bedouin-Muslims origin and attitudes toward BMD screening highlights the need to better understand ethno-cultural backgrounds when investigating cognitions and motivations related to health behaviors. Our findings contribute to a more comprehensive understanding of how background variables, culture, and cognitions affect the performance of preventive screening behaviors.

The low level of knowledge about osteoporosis and BMD screening behavior among the study groups elucidates the need for health promotion interventions and initiatives. One of the implications of our study is that, in order to assure sufficient BMD screening behavior among all ethno-cultural groups, intervention programs—tailored to address the unique characteristics of each ethno-cultural group—are required, together with educational programs for physicians, nurses, and other allied health professionals working in the community.

## Figures and Tables

**Table 1:** Descriptive statistics of the study variables by ethnicity. FSU—Former Soviet Union.

Ethno-Cultural Groups					
Total Sample	Israeli-Born Jews	FSU Jewish Immigrants	Israeli-Born Bedouin-Muslims	F/χ^2^	Total Sample
	(*n* = 300)	(*n* = 100)	(*n* = 100)	(*n* = 100)	
	Mean (SD)/%	Mean (SD)/%	Mean (SD)/%	Mean (SD)/%	
**Age**	70.9 (7.1)	68.2 (6.4)	70.3 (6.7)	74.2 (6.8) ^ab^	20.91 **^ǂ^
**Religiosity**					125.79 **^§^
Secular	36.1	28.3	77.0	1.1	
Traditional	63.9	71.7	23.3	98.9	
**Marital status**					4.43 **^§^
Married or cohabitating	64.8	73.2	60.6	60.8	
Other	35.2	26.8	39.4	39.2	
**Number of Children**	3.81 (2.26)	3.48 (1.75) ^b^	2.14 (.90) ^a^	5.73 (2.19) ^ab^	207.34 **^§^
0–2	30.2	17.2	75.0	0.0	
3–4	42.4	69.7	21.9	35.0	
5 and above	27.5	13.1	3.1	65.5	
**Education**					129.94 **^§^
Low	57.3	47.7	23.5	100	
Medium	17.3	26.8	25.5	0	
High	25.4	25.8	51.0	0	
**Perceived financial situation**					70.70 **^§^
Below Average	30.0	11.1	50.0	29.0	
Average	40.4	61.6	38.8	21.0	
Above Average	29.6	27.3	11.2	50.0	
**Employment**					51.01 **^§^
Yes	28.9	46.5	37.4	3.0	
No	71.1	53.3	62.6	97.0	

Note. ** *p* < 0.01. ^a^ Significantly different (*p* < 0.001) Israeli-born Jews (Bonferroni post hoc comparisons). ^b^ Significantly different (*p* < 0.001) FSU Jewish immigrants (Bonferroni post hoc comparisons). ^ǂ^ Denotes ANOVA’s F value; ^§^ denotes χ^2^ test.

**Table 2:** Differences in knowledge about osteoporosis and theory of planned behavior (TPB) components by ethnicity (ANOVA). BMD—bone mineral density.

Ethno-Cultural Groups					
	Total Sample	Israeli-Born Jews	FSU Jewish Immigrants	Israeli-Born Bedouin-Muslims	F
	(*n* = 300)	(*n* = 100)	(*n* = 100)	(*n* = 100)	
	Mean (SD)/%	Mean (SD)/%	Mean (SD)/%	Mean (SD)/%	
Knowledge about osteoporosis (0–24)	9.51(3.88)	10.38(4.32)	10.74(3.73)	7.43(2.49) ^ab^	25.44 **
Perceived control regarding BMD screening (2–14)	11.30(3.02)	13.14(1.85) ^b^	11.88(3.09) ^a^	8.89(2.24) ^ab^	79.13 **
Attitudes toward BMD screening (2–14)	10.93(2.99)	11.74(2.39)	11.43(3.04)	9.64(3.06) ^ab^	15.77 **
Subjective norms regarding BMD screening (2–14)	10.17(3.18)	10.86(2.92)	10.40(3.91)	9.26(2.30) ^a^	6.97 **
Intention to undergo BMD screening (1–7)	5.86(1.43)	6.27(1.28)	5.87(1.48)	5.43(1.40) ^a^	8.97 **

Note. ** *p* < 0.01. ^a^ Significantly different (*p* < 0.001) Israeli-born Jews (Bonferroni post hoc comparisons). ^b^ Significantly different (*p* < 0.001) FSU Jewish immigrants (Bonferroni post hoc comparisons).

**Table 3:** Bivariate analysis of the study variables by BMD screening behavior (*n* = 300).

BMD Screening Behavior			
	Yes 58.7% (*n* = 176)	No 41.3% (*n* = 124)	*t*-Test/χ^2^
	Mean (SD)/%	Mean (SD)/%	
Age	72.74 (7.16)	69.25 (6.77)	4.19 **^‡^
Religiosity			6.29 *^§^
Secular	34.9	65.1	
Traditional and religious	50.3	49.7	
Marital status			2.28 ^§^
Married or cohabitating	47.0	53.0	
Other	37.5	62.5	
Number of Children			1.91 ^§^
0–2	42.0	58.0	
3–4	40.8	59.2	
5 and above	50.7	49.3	
Education			5.33 ^§^
Low	49.4	50.6	
Medium	42.0	58.0	
High	33.3	66.7	
Perceived financial situation			7.51 *^§^
Below Average	44.6	55.4	
Average	35.9	64.1	
Above Average	55.6	44.4	
Employment			5.49 *^§^
Yes	33.3	66.7	
No	48.5	51.5	
Ethnicity			6.89 *^§^
Israeli-born Jews	50.5	49.5	
FSU Jewish immigrants	33.3	66.7	
Israeli-born Bedouin-Muslims	48.2	51.8	
Knowledge about osteoporosis (0–24)	10.20 (3.59)	9.37 (3.98)	1.8 ^*‡*^
Perceived control regarding BMD screening (2–14)	11.91 (2.45)	11.18 (3.20)	2.16 *^‡^
Attitudes toward BMD screening (2–14)	11.89 (1.99)	10.40 (3.35)	4.63 **^‡^
Subjective norms regarding BMD screening (2–14)	11.16 (2.46)	9.53 (3.43)	4.65 **^‡^
Intention to perform BMD screening (1–7)	6.44 (0.83)	5.44 (1.61)	6.97 **^‡^

Note. * *p* < 0.05; ** *p* < 0.01; ^*‡*^ denotes *t*-test; ^§^ denotes χ^2^ test.

**Table 4:** Logistic regressions: Prediction of BMD screening behavior (*n* = 300).

BMD Screening ^(a)^										
	Model 1		Model 2		Model 3		Model 4		Model 5	
	B (SE)	OR	B (SE)	OR	B (SE)	OR	B (SE)	OR	B (SE)	OR
Age	0.10 ** (0.02)	1.11	0.10 ** (0.02)	1.11	0.11 ** (0.02)	1.12	0.11 ** (0.02)	1.12	0.11 ** (0.02)	1.12
Ethnicity										
Israeli-born Jews (ref)										
FSU Jewish immigrants	−0.84 * (0.37)	0.43	−0.88 * (0.37)	0.41	−0.75 (0.39)	0.47	−0.70 (0.41)	0.49	−0.36 (2.01)	0.26
Israeli-born Bedouin-Muslims	−1.30 ** (0.41)	0.27	−1.05 * (0.43)	0.34	−0.38 (0.52)	0.68	−0.72 (0.56)	0.48	−0.48 * (2.05)	0.08
Marital status										
Other (ref)										
Married or cohabitating	0.97 ** (0.30)	2.65	0.87 ** (0.31)	2.40	0.91 ** (0.33)	2.50	0.98 ** (0.34)	2.68	1.02 ** (0.35)	2.78
Number of Children	−0.03 (0.07)	0.96	−0.03 (0.07)	0.96	−0.01 (0.08)	0.99	0.01 (0.08)	1.01	0.03 (0.08)	1.03
Education										
Low (ref)										
Medium	−0.05 (0.40)	0.95	−0.09 (0.41)	0.91	−0.11 (0.43)	0.89	−0.15 (0.45)	0.85	−0.16 (0.44)	0.84
High	−0.57 (0.38)	0.56	−0.73 (0.39)	0.48	−0.72 (0.41)	0.48	−0.84 (0.44)	0.43	−0.83 (0.43)	0.43
Perceived financial situation										
Below Average (ref)										
Average	−0.47 (0.35)	0.62	−0.43 (0.35)	0.64	−0.35 (0.38)	0.70	−0.28 (0.39)	0.75	−0.26 (0.40)	0.76
Above Average	0.23 * (0.36)	1.27	0.18 (0.36)	1.20	0.31 (0.39)	1.37	0.44 (0.41)	1.56	0.46 (0.42)	1.58
Knowledge about osteoporosis (0–24)			0.10 * (0.03)	1.10	0.07 (0.04)	1.07	0.09 * (0.04)	1.10	0.10 * (0.04)	1.10
Perceived control regarding BMD screening (2–14)					0.07 (0.07)	1.07	−0.05 (0.08)	0.94	−0.07 (0.08)	0.92
Attitudes toward BMD screening (2–14)					0.19 ** (0.06)	1.21	0.14 * (0.07)	1.15	−0.14 (0.06)	0.94
Subjective norms regarding BMD screening (2–14)					0.13 * (0.05)	1.14	0.09 (0.05)	1.09	0.11 * (0.06)	1.11
Intention to perform BMD screening (1–7)							0.65 ** (0.16)	1.92	0.64 ** (0.16)	1.91
Immigrants × Attitudes									0.24 (0.16)	1.27
Bedouin-Muslims × Attitudes									0.36 * (0.17)	1.43
Intercept	−7.36 ** (1.67)		−8.38 ** (1.75)		−13.50 ** (2.29)		−15.52 ** (2.51)		−13.21 ** (2.77)	
Chi-square	44.43 **		51.23 **		81.44 **		103.23 **		108.18 **	
Pseudo *R^2^*	0.188		0.214		0.324		0.396		0.412	

^(a)^ Reference group consists of participants who did not perform the test. OR = odds ratio. Note. * *p* < 0.05; ** *p* < 0.01.
